# Long non-coding RNA TUG1 promotes proliferation and migration in PDGF-BB-stimulated HASMCs by regulating miR-216a-3p/SMURF2 axis

**DOI:** 10.1186/s12860-021-00396-0

**Published:** 2021-11-08

**Authors:** Xinfang Wang, Junsong Chen

**Affiliations:** 1 Department of Pediatrics, Hangzhou First People’s Hospital Affiliated to Zhejiang University, Zhejiang, Hangzhou China; 2 Respiratory Department, Hangzhou Children’s Hospital, 195 Wenhui Road, Zhejiang, 310003 Hangzhou China

**Keywords:** Childhood asthma, TUG1, miR-216a-3p, SMURF2, HASMCs

## Abstract

**Background:**

Abnormal proliferation and migration of human airway smooth muscle cells (HASMCs) play an important role in the development of childhood asthma. Long non-coding RNAs (lncRNAs) have been demonstrated to participate in HASMC proliferation and migration. We aimed to explore more effects and molecular mechanism of taurine upregulated gene 1 (TUG1) in childhood asthma.

**Results:**

TUG1 and SMURF2 were overexpressed and miR-216a-3p was downregulated in childhood asthma patients and PDGF-BB-stimulated HASMCs. TUG1 knockdown attenuated PDGF-BB-triggered proliferation and migration of HASMCs. MiR-216a-3p was targeted by TUG1, and miR-216a-3p suppression counteracted the repressive effects of TUG1 interference on proliferation and migration in PDGF-BB-treated HASMCs. SMURF2 was a downstream target of miR-216a-3p, and SMURF2 upregulation abated the inhibiting effects of miR-216a-3p on migration and proliferation in PDGF-BB-exposed HASMCs. TUG1 sponged miR-216a-3p to positively regulate SMURF2 expression.

**Conclusion:**

TUG1 downregulation inhibited PDGF-BB-induced HASMC proliferation and migration by regulating miR-216a-3p/SMURF2 axis, offering novel insight into the potential application of TUG1 for childhood asthma treatment.

**Supplementary Information:**

The online version contains supplementary material available at 10.1186/s12860-021-00396-0.

## Background

Childhood asthma is a group of multifactorial diseases characterized by wheezing, coughing, chest tightness, dyspnoea, inflammation, airway remodeling, and airway hyper-responsiveness [10, 21]. The prevalence of childhood asthma is increasing worldwide [22]. Although enormous efforts have been made to improve the treatment of childhood asthma, it is still difficult to effectively control childhood asthma due to the heterogenicity and complexity of childhood asthma [3]. Human airway smooth muscle cells (HASMCs) play a pivotal role in the multiple biological processes, and excessive proliferation and migration of HASMCs directly accelerate the development of childhood asthma [15, 20]. Thus, it is critical to explore the underlying mechanisms that regulate the proliferation and migration of HASMCs.

Long non-coding RNAs (lncRNAs) are a type of non-coding RNA (ncRNAs) and involved in diverse physiopathology processes [8, 16, 34]. In recent years, many reports have indicated that lncRNAs are pivotal regulators in cell behaviors and the progression of many diseases, including asthma [23]. For example, lncRNA TCF7 accelerated the migration and proliferation of airway smooth muscle cells (ASMCs) by regulating TIMMDC1/Akt axis in asthma [7]. In addition, lncRNA GAS5 contributed to the proliferation of ASMCs via targeting miR-10a/BDNF signaling pathway [35]. TUG1, a 6.7 kilobase (kb) RNA sequence, played critical roles in asthma [18]. Nevertheless, the precise mechanism by which TUG1 influences childhood asthma is not completely understood.

Th competitive endogenous RNA (ceRNA) networks hypothesis has been proposed in a variety of diseases, which indicates lncRNA can serve as microRNA (miRNA) sponge or decoy to modulate the expression of miRNA targets [25]. MiRNAs are usually small ncRNAs that can modulate target gene expression [1, 13]. A previous report demonstrated that miR-216a level was considerably increased in ASMCs of asthmatic patients [29]. Nevertheless, the biological functions and regulatory mechanism of miR-216a-3p in childhood asthma are still poorly defined.

SMURF2 (Smad ubiquitin protein ligase 2) is an ubiquitin ligase for Smads that regulates TGF-β signaling pathway via ubiquitin-proteasome pathway (UPP) [28]. A previous study declared that SMURF2 knockdown repressed ASMCs proliferation and increased apoptosis in mice with chronic asthma [27]. However, the regulatory mechanisms of SMURF2 in childhood asthma pathogenesis have not been thoroughly elucidated.

In this study, PDGF-BB was applied to induce HASMC proliferation and migration. Moreover, we examined TUG1, miR-216a-3p and SMURF2 expression in blood samples of patients with childhood asthma and PDGF-BB-stimulated HASMCs. Additionally, the biological roles of TUG1, miR-216a-3p and SMURF2 on the proliferation and migration of HASMCs were explored. The TUG1/miR-216a-3p/SMURF2 axis was proposed to provide a novel theoretical basis for childhood asthma treatment.

## Methods

### Blood collection and RNA isolation

The whole blood samples were obtained from Hangzhou First People’s Hospital Affiliated to Zhejiang University between March 2019 and October 2019. All participants did not receive chemotherapy or radiotherapy. Childhood asthma blood samples were obtained from 32 patients with childhood asthma. The patients were diagnosed according to Global Initiative for Asthma (GINA) guideline [9]. Control subjects were from 32 age- and sex-matched healthy volunteers. Participants with abnormal liver function, chronic bronchitis, tuberculosis, pulmonary embolism, coinfection, and blood system diseases were excluded. This study was approved by the ethics committee of Hangzhou First People’s Hospital Affiliated to Zhejiang University, and written informed consent was acquired from every participant.

### Cell culture and transfection

HASMCs were purchased from American Tissue Culture Collection (Manassas, VA, USA) and cultured in DMEM (Invitrogen, Carlsbad, CA, USA) containing 10% FBS (Gibco, Carlsbad, CA, USA) at 37 °C under a humidified air with 5% CO_2_. To construct childhood asthma model in vitro, HASMCs were exposed to PDGF-BB (20 ng/mL) (Peprotech, Rocky Hill, NJ) for 12 h.

The siRNA against TUG1 (si-TUG1) and matched control (si-NC), miR-216a-3p mimic or inhibitor (miR-216a-3p or anti-miR-216a-3p) and matched control (miR-NC or anti-miR-NC), SMURF2-overexpressing plasmid (SMURF2) and matched control (pcDNA) were commercially acquired from Genecreat (Wuhan, China). The sequences were as follows: si-TUG1 (sense, 5′-UACUGUUUCUUUAAAUGGCGG-3′, antisense, 5′-GCCAUUUAAAGAAACAGUACC-3′); si-NC (sense, 5′- UUCUCCGAACGUGUCACGUTT-3′, antisense, 5′-ACGUGACACGUUCGGAGAATT-3″); miR-216a-3p mimic (5′- UCACAGUGGUCUCUGGGAUUAU-3′); miR-NC (5′-UUCUCCGAACGUGUCACGUTT-3′); miR-216a-3p inhibitor (5′-AUAAUCCCAGAGACCACUGUGA-3′); anti-miR-NC (5′-CAGUACUUUUGUGUAGUACAA-3′). For cell transfection, HASMCs were introduced with the above oligonucleotide or/and vector using HiPerFect transfection reagent (Qiagen, Valencia, CA, USA).

### Quantitative real-time PCR (qRT-PCR)

TRIzol reagent (Invitrogen) was employed to isolate the total RNA from HASMCs. The first strand of cDNA was synthesized using the Prime Script RT reagent Kit (TaKaRa, Kusatsu, Japan) for analysis TUG1 and SMURF2, or using miScript II RT Kit (Qiagen) for detection of miR-216a-3p. Next, the diluted cDNA was subjected to qRT-PCR using SYBR Green PCR Kit (Takara) on ABI 7500 Real-time PCR system (Thermo Fisher Scientific, Waltham, MA, USA). The RNA levels were calculated via 2^-ΔΔCt^ method. GAPDH and U6 were acted as the internal references for TUG1, SMURF2 and miR-216a-3p, respectively. The following primers were used for qRT-PCR: TUG1 (forward 5′-3′: CTGAAGAAAGGCAACATC; reverse 5′-3′: GTAGGCTACTACAGGATTTG) miR-216a-3p (forward 5′-3′: GCCGAGTCACAGTGGTCTCT; reverse 5′-3′: CAGTGCGTGTCGTGGAGT), SMURF2 (forward 5′-3′: TCCTCGGCTGTCTGCTAACTTG; reverse 5′-3′: CAGGCATTCTGTGTCATCAGGAC), GAPDH (forward 5′-3′: ACCCACTCCTCCACCTTTGAC; reverse 5′-3′: TGTTGCTGTAGCCAAATTCGTT), U6 (forward 5′-3′: CTCGCTTCGGCAGCACATATACT; reverse 5′-3′: ACGCTTCACGAATTTGCGTGTC).

### Cell viability assay

HASMCs were inoculated into 96-well plates. Following treatment, 10 μL of Cell Counting Kit-8 (CCK-8; Boster, Wuhan, China) solution was placed into per well for 2–3 h at 37 °C, followed by detection of the absorbance using a microplate reader (Thermo Fisher Scientific) at 450 nm wavelength .

### Cell cycle assay

HASMCs were collected after treatment for 48 h, and fixed using ice-cold 70% ethanol at − 20 °C for 12 h. Afterwards, HASMCs were collected and washed with PBS, and suspended in PBS that contained propidium iodide (PI; 25 μg/mL) solution, 0.2% (v/v) Triton X-100 and 20 μg/mL DNase-free RNase. At last, cell cycle distribution was detected using flow cytometry (Partec AG, Arlesheim, Switzerland).

### Transwell assay

Migration of HASMCs was evaluated using transwell chamber. Briefly, HASMCs were resuspended in serum-free medium (100 μL) and added into the top chamber. The medium containing 10% FBS (500 μL) was placed into the lower part of the chamber. 24 h later, HASMCs remaining on the top membrane surface were gently removed, and HASMCs migrated to the lower chamber were fixed in 95% ethanol, followed by staining with 0.1% crystal violet solution. At last, the migrated cells were photographed and counted using a microscope (100× magnification).

### Bioinformatics analysis and dual-luciferase reporter assay

The relationship between miR-216a-3p and TUG1 or SMURF2 was predicted using starBase v2.0 (http://starbase.sysu.edu.cn/). TUG1 or 3’UTR of SMURF2 including putative miR-216a-3p target binding sequence was amplified and respectively cloned into the pmirGlO luciferase reporter vector (Promega, Madison, WI, USA) to create WT-TUG1 and SMURF2 3’UTR-WT. Meanwhile, the binding sequence for miR-216a-3p was mutated and inserted into the same vector to construct MUT-TUG1 and SMURF2 3’UTR-MUT. Next, HASMCs were co-introduced with miR-216a-3p/miR-NC and WT/MUT luciferase reporter plasmid for 48 h. At last, the luciferase activity was measured by dual-Luciferase Reporter Assay System (Promega).

### Western blot assay

RIPA lysis buffer (Thermo Fisher Scientific) was used for isolating total protein. After measurement of protein concentration, approximately 40 μg of extracted protein was separated by SDS-PAGE, and then transferred (semi-dry method) onto PVDF membrane. The membranes were blocked and then probed with primary antibodies against SMURF2 (ab94483, 1:1000, Abcam, Cambridge, UK) or GAPDH (ab37168, 1:1000, Abcam) at 4°Cfor 12–14 h. After incubation with secondary antibody (D110058, 1:4000, Sangon Biotech, Shanghai, China), the combined signals were visualized using ECL reagent (Tanon, Shanghai, China). The band density was assessed by ImageJ software.

### Statistical analysis

All data were shown as mean ± standard deviation and all experiments were repeated at least three times. For comparison within different groups, two-tailed Student’s *t*-test and a one-way analysis of variance (ANOVA) were performed. The correlation analysis between miR-216a-3p and TUG1 or SMURF2 was performed by Spearman rank correlation. Statistical analyses were performed using GraphPad Prism 7.0. Statistical significance was considered when *P* < 0.05.

## Results

### TUG1 was overexpressed in patients with childhood asthma and PDGF-BB-stimulated HASMCs

**Fig. 1:**
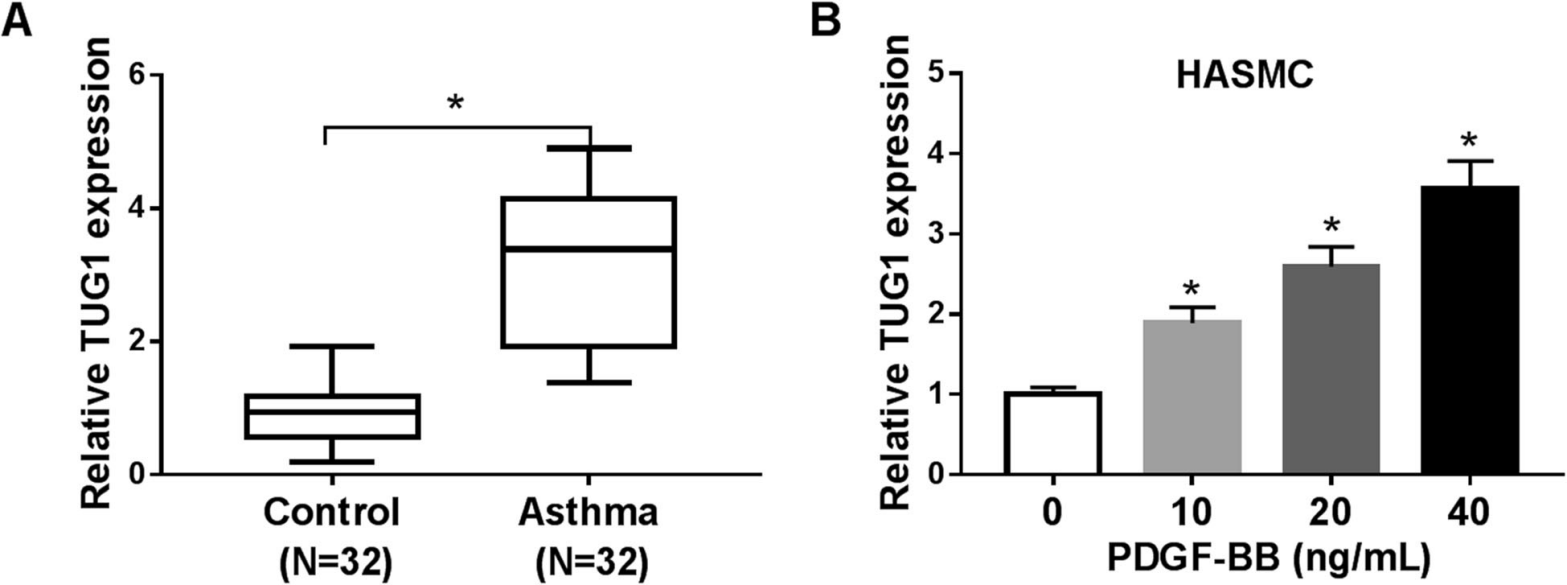
TUG1 level was increased in childhood asthma patients and PDGF-BB-stimulated HASMCs. **A** TUG1 expression was measured by qRT-PCR in whole blood samples of childhood asthma patients (*N* = 32) and healthy subjects (N = 32) (*t*-test). **B** The level of TUG1 was determined by qRT-PCR in HASMCs exposed to different concentrations of PDGF-BB (ANOVA). *P < 0.05

To assess whether TUG1 was involved in the pathogenesis of asthma, the expression of TUG1 was detected. The level of TUG1 was enhanced 3.34-fold in whole blood samples of childhood asthma patients relative to healthy controls (Fig. 1A). Similarly, TUG1 expression was dose-dependently increased in HASMCs treated with PDGF-BB (Fig. 1B). Moreover, we found the concentrations of IL-4, IL-5, and IL-13 were increased 4.71-fold, 3.58-fold and 3.42-fold in serum samples of asthma patients compared to healthy controls, respectively (Additional file 1). Our data indicated that TUG1 might play a critical role in childhood asthma.

### Knockdown of TUG1 inhibited PDGF-BB-induced HASMC cell proliferation and migration

**Fig. 2:**
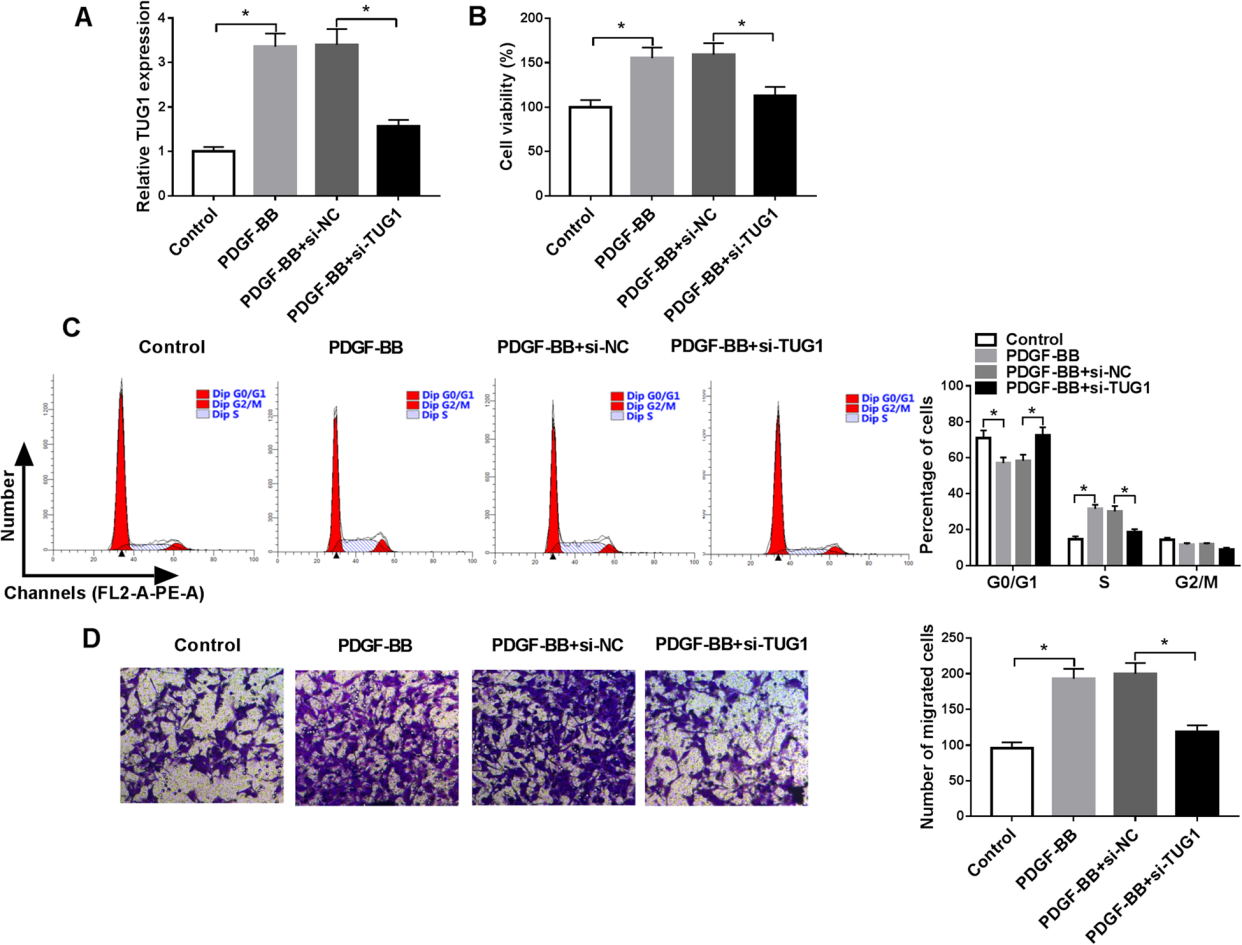
TUG1 interference attenuated PDGF-BB-caused HASMCs cell proliferation and migration. HASMCs were divided into four groups: Control, PDGF-BB (20 ng/mL), PDGF-BB + si-NC, and PDGF-BB + si-TUG1. **A** TUG1 expression was examined (ANOVA). (B) CCK-8 assay was used for measuring cell viability (ANOVA). **C** Cell cycle distribution was examined via flow cytometry (ANOVA). **D** Cell migration was evaluated using transwell assay (100×) (ANOVA). *P < 0.05

To explore the impact of TUG1 on HASMC proliferation and migration in under stimulation with PDGF-BB, knockdown of TUG1 was constructed using the siRNA. TUG1 expression was increased 3.35-fold by treatment with PDGF-BB, which was reversed by knockdown of TUG1 (Fig. 2A). Moreover, HASMC viability was increased 1.55-fold by PDGF-BB stimulation, whereas interference of TUG1 inhibited PDGF-BB-induced cell viability (Fig. 2B). The ratio of HASMCs in G0/G1 phase was decreased 19.71% by treatment with PDGF-BB and the ratio of HASMCs in S phase was elevated 2.16-fold (Fig. 2C), suggesting PDGF-BB-induced HASMCs to enter the synthesis phase (S phase) for proliferation. However, downregulation of TUG1 abated the effect of PDGF-BB on cell cycle progression via increasing the ratio of HASMCs in G0/G1 phase and reducing the ratio of HASMCs in S phase (Fig. 2C). Transwell assay indicated that migration of HASMCs was increased after exposure to PDGF-BB, which was abolished by downregulating TUG1 (Fig. 2D). Collectively, these data suggested that TUG1 might be involved in childhood asthma progression.

### TUG1 could bind to miR-216a-3p

**Fig. 3:**
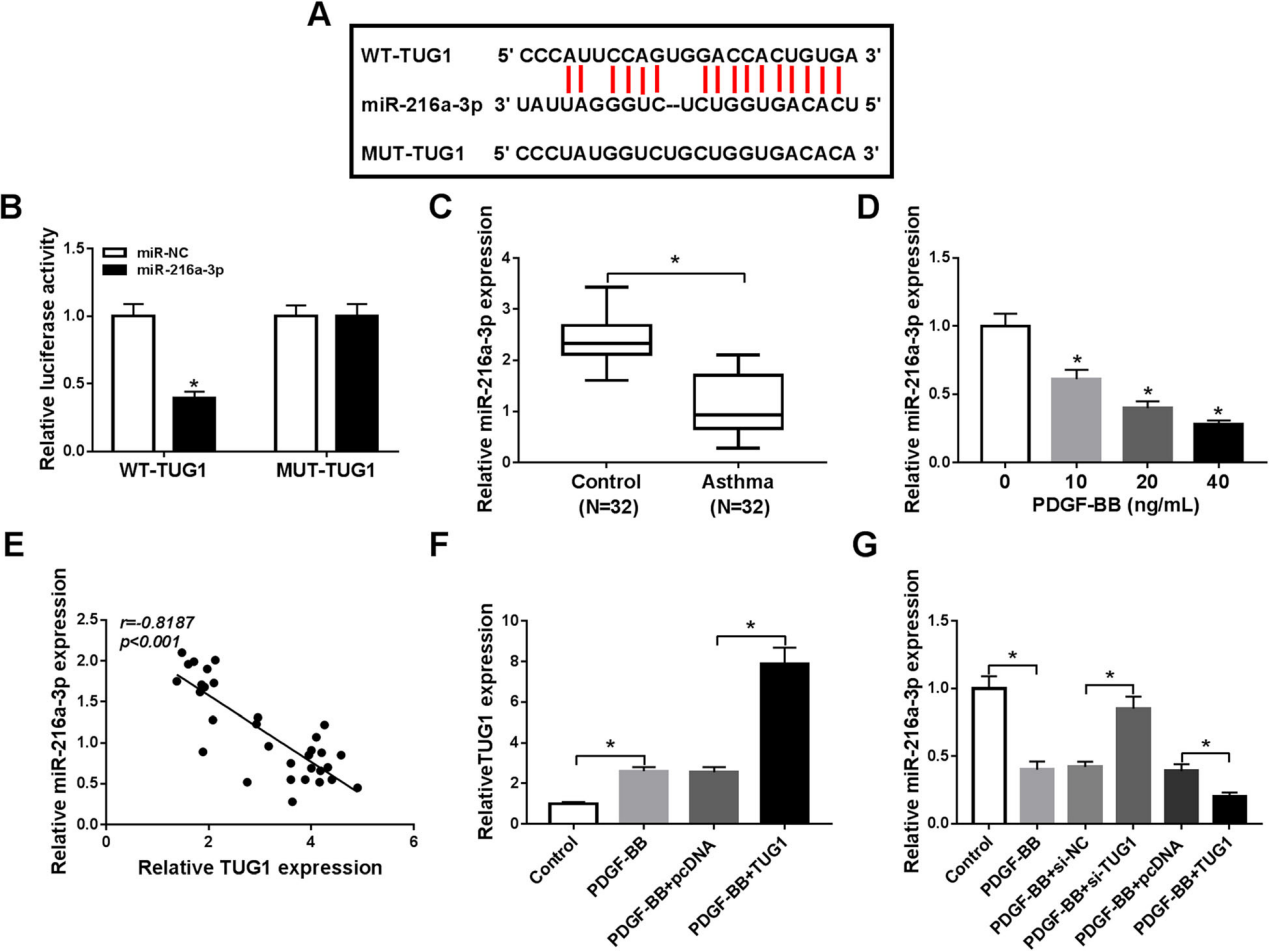
TUG1 served as a miR-216a-3p sponge in HASMCs**. A** StarBase v2.0 predicted the complementary sequences between TUG1 and miR-216a-3p. **B** The luciferase activity was measured in HASMCs co-introduced with WT/MUT-TUG1 and miR-216a-3p/miR-NC using the dual-luciferase luciferase reporter assay (ANOVA). **C** MiR-216a-3p expression was measured in whole blood samples of childhood asthma patients and healthy subjects (*t*-test). **D** MiR-216a-3p expression was examined in HASMCs stimulated with different concentrations of PDGF-BB (ANOVA). **E** The correlation between TUG1 level and miR-216a-3p expression was analyzed in whole blood samples of childhood asthma patients. **F** TUG1 expression was determined in HASMCs (Control) or HASMCs treated with PDGF-BB, PDGF-BB + pcDNA, or PDGF-BB + TUG1 (ANOVA). **G** The level of miR-216a-3p was examined in HASMCs or HASMCs treated with PDGF-BB, PDGF-BB + si-NC, PDGF-BB + si-TUG1, PDGF-BB + pcDNA, or PDGF-BB + TUG1 (ANOVA). *P < 0.05

To elucidate the underlying mechanism by which TUG1 regulated the HASMC proliferation and migration, starBase v2.0 was used. There were binding sites between miR-216a-3p and TUG1 (Fig. 3A). For confirming the interaction between TUG1 and miR-216a-3p, dual-luciferase reporter analysis was performed. WT-TUG1 luciferase activity was decreased 61% by transfection with miR-216a-3p, (Fig. 3B). However, the luciferase activity of MUT-TUG1 was unaffected after introduction with miR-216a-3p (Fig. 3B). Subsequently, miR-216a-3p expression in childhood asthma patients and PDGF-BB-stimulated HASMCs was analyzed. MiR-216a-3p expression was reduced 52.55% in whole blood samples of childhood asthma patients compared to healthy controls (Fig. 3C). Likewise, miR-216a-3p level was also dose-dependently decreased in HASMCs exposed to PDGF-BB (Fig. 3D). And we observed that there was inversive correlation between miR-216a-3p level and TUG1 expression in whole blood samples of childhood asthma patients (Fig. 3E). TUG1 was overexpressed in PDGF-BB-stimulated HASMCs transfected with TUG1 (Fig. 3F). TUG1 deficiency increased miR-216a-3p expression, whereas upregulation of TUG1 decreased miR-216a-3p expression (Fig. 3G). All these findings manifested that miR-216a-3p was targeted by TUG1.

### MiR-216a-3p knockdown reversed the inhibitory impact of si-TUG1 on the proliferation and migration of HASMCs under stimulation with PDGF-BB

**Fig. 4:**
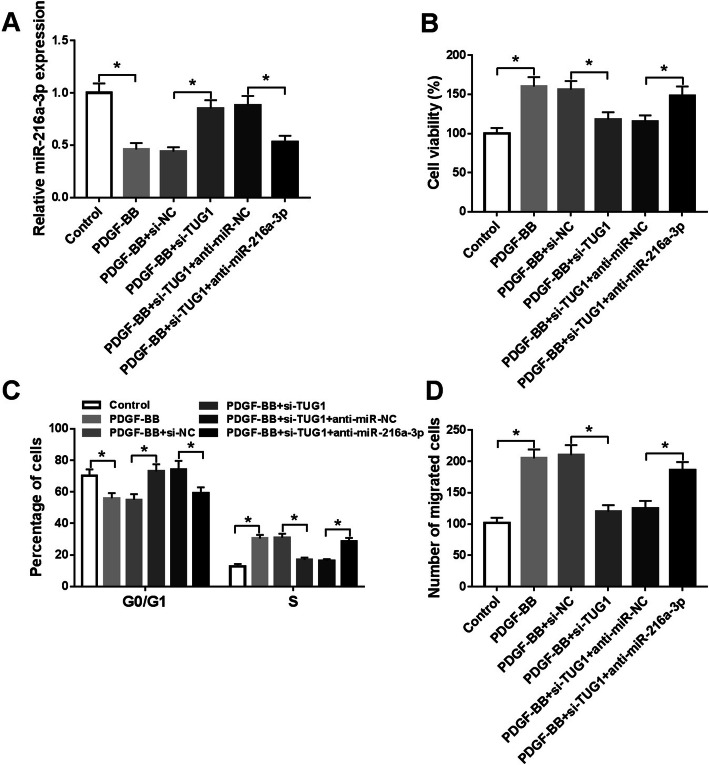
TUG1 knockdown inhibited the proliferation and migration by upregulating miR-216a-3p in PDGF-BB-stimulated HASMCs. HASMCs were divided into six groups: Control, PDGF-BB, PDGF-BB + si-NC, PDGF-BB + si-TUG1, PDGF-BB + si-TUG1 + anti-miR-NC, and PDGF-BB + si-TUG1 + anti-miR-216a-3p. **A** The level of miR-216a-3p was measured (ANOVA). **B**-**D** Cell viability, cell cycle progression, and migration were assessed (ANOVA). (ANOVA). *P < 0.05

To clarify whether TUG1 exerted its roles through sponging miR-216a-3p, rescued experiments were performed in PDGF-BB-exposed HASMCs. Knockdown of TUG1 increased miR-216a-3p abundance, which was inhibited by downregulating miR-216a-3p in PDGF-BB-stimulated HASMCs (Fig. 4A). Additionally, miR-216a-3p inhibition expression neutralized the suppressive impact of TUG1 silencing on cell viability (Fig. 4B). Furthermore, the enhanced G0/G1 phase cells and reduced S phase cells caused by TUG1 knockdown were reversed after downregulating miR-216a-3p (Fig. 4C). Besides, the suppressive influence of si-TUG1 on cell migration was also abolished after interference of miR-216a-3p in PDGF-BB-induced HASMCs (Fig. 4D). Taken together, TUG1 regulated HASMC proliferation and migration under treatment with PDGF-BB by sponging miR-216a-3p.

### SMURF2 was a direct target of miR-216a-3p

**Fig. 5:**
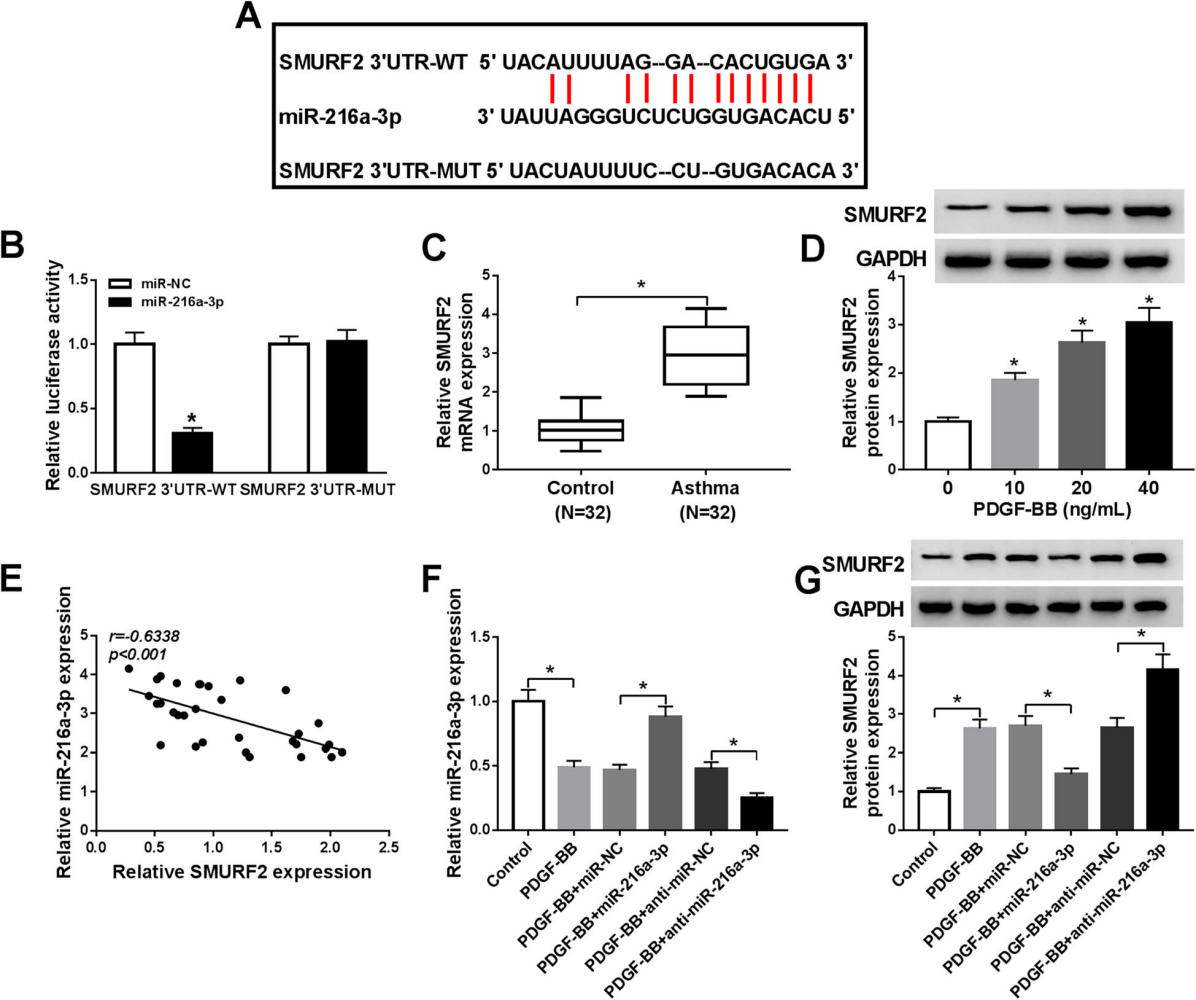
SMURF2 was targeted by miR-216a-3p in HASMCs. **A** The potential binding sites of miR-216a-3p and SMURF2 were predicted by starBase v2.0. **B** Relative luciferase activity was determined in HASMCs co-introduction with SMURF2 3’UTR-WT/MUT and miR-216a-3p/miRNA-NC (ANOVA). **C** SMURF2 mRNA expression was determined by qRT-PCR in whole blood samples of childhood asthma patients (*t*-test). **D** SMURF2 protein expression was measured using western blot assay in HASMCs exposed to different concentrations of PDGF-BB (ANOVA). The blots have been cropped. **E** The correlation between SMURF2 level and miR-216a-3p expression was analyzed in whole blood samples of childhood asthma patients. **F** and **G** MiR-216a-3p expression and SMURF2 protein expression were examined in HASMCs or HASMCs treated with PDGF-BB, PDGF-BB + miR-NC, PDGF-BB + miR-216a-3p, PDGF-BB + anti-miR-NC, or PDGF-BB + anti-miR-216a-3p (ANOVA). The blots have been cropped. *P < 0.05

To probe the regulatory mechanism of miR-216a-3p, we screened the target mRNAs of miR-216a-3p using starBase v2.0. The results presented that miR-216a-3p and SMURF2 3’UTR had complementary binding sites (Fig. 5A), implying that SMURF2 might be targeted by miR-216a-3p. SMURF2 3’UTR-WT luciferase activity was declined 69% in HASMCs transfected with miR-216a-3p, but luciferase activity of SMURF2 3’UTR-MUT was not affected after introduction with miR-216a-3p (Fig. 5B). The mRNA expression of SMURF2 was increased 2.71-fold in whole blood samples of childhood asthma patients relative to healthy controls (Fig. 5C). Likewise, SMURF2 protein expression was also dose-dependently enhanced in HASMCs exposed to PDGF-BB (Fig. 5D). Moreover, miR-216a-3p abundance was negatively correlated with SMURF2 mRNA expression in childhood asthma patients (Fig. 5E). Furthermore, miR-216a-3p expression was enhanced by transfection of miR-216a-3p and decreased by transfection of anti-miR-216a-3 (Fig. 5F), suggesting miR-216a-3p and anti-miR-216a-3p were successfully introduced into PDGF-BB-stimulated HASMCs. Enforced expression of miR-216a-3p reduced the level of SMURF2 protein, while knockdown of miR-216a-3p promoted SMURF2 protein expression in HASMCs exposed to PDGF-BB (Fig. 5G). Altogether, these data implicated that miR-216a-3p could bind to SMURF2 and negatively regulated SMURF2 expression.

### MiR-216a-3p overexpression inhibited cell proliferation and migration in HASMCs stimulated with PDGF-BB via reducing SMURF2 expression

**Fig. 6:**
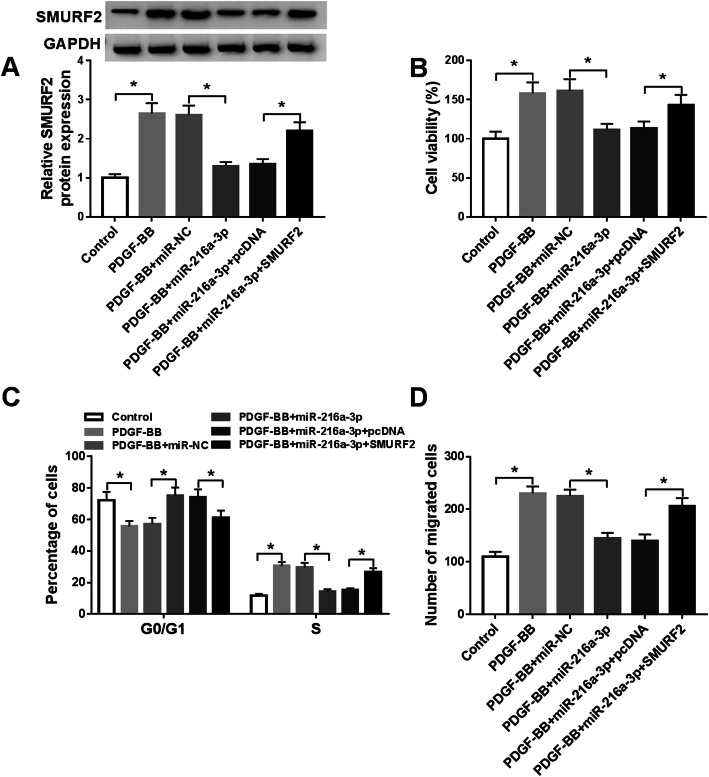
Overexpression of miR-216a-3p repressed cell proliferation and migration in PDGF-BB-stimulated via downregulating SMURF2. HASMCs were divided into six groups: Control, PDGF-BB, PDGF-BB + miR-NC, PDGF-BB + miR-216a-3p, PDGF-BB + miR-216a-3p + pcDNA, and PDGF-BB + miR-216a-3p + SMURF2. **A** The protein abundance of SMURF2 was analyzed (ANOVA). The blots have been cropped. **B**-**D** Cell viability, cell cycle distribution, and migration were examined (ANOVA). *P < 0.05

To investigate whether SMURF2 participated in miR-216a-3p-mediated functions, HASMCs were introduced with miR-NC, miR-216a-3p, miR-216a-3p + pcDNA, or miR-216a-3p + SMURF2, followed by treatment with PDGF-BB. Restoration of miR-216a-3p led to a decrease in the protein expression of SMURF2, which was rescued by addition of SMURF2 (Fig. 6A). CCK-8 analysis revealed that miR-216a-3p overexpression reduced cell viability in PDGF-BB-stimulated HASMCs, while upregulation of SMURF2 abated this effect (Fig. 6B). Upregulation of miR-216a-3p could increase the ratio of HASMCs in G0/G1 phase and decrease the ratio of HASMCs in S phase, whereas this effect was reversed by upregulating SMURF2 (Fig. 6C). Moreover, the anti-migration effect caused by miR-216a-3p was also restored by co-transfection of SMURF in HASMCs exposed to PDGF-BB (Fig. 6D). Our findings indicated that miR-216a-3p exerted its biological functions by targeting SMURF2 in PDGF-BB-stimulated HASMCs.

### TUG1 regulated SMURF2 expression by sponging miR-216a-3p

**Fig. 7:**
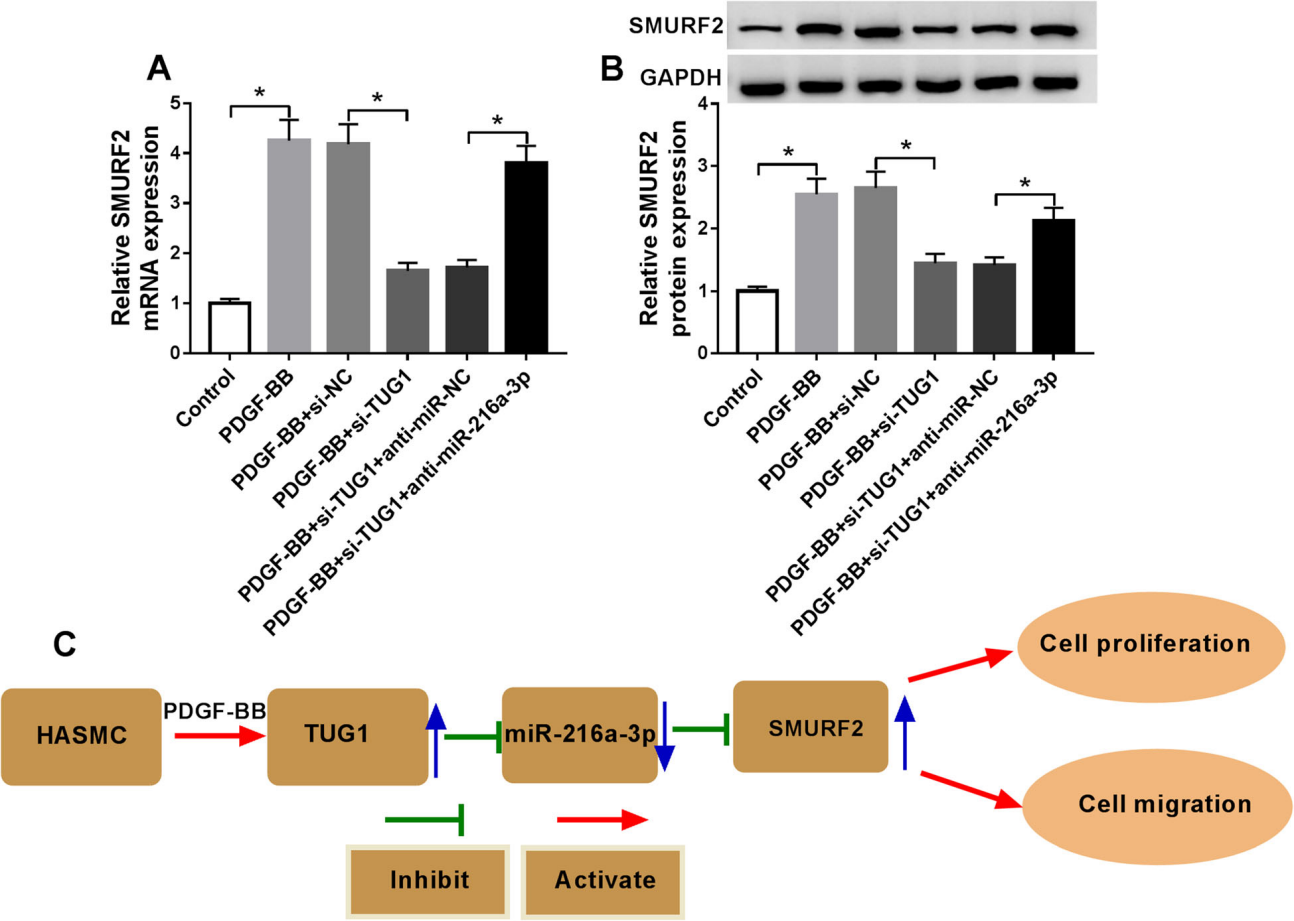
TUG1 modulated SMURF2 expression via sponging miR-216a-3p. **A** and **B** The mRNA level and protein level of SMURF2 were detected in HASMCs or HASMCs treated with PDGF-BB, PDGF-BB + si-NC, PDGF-BB + si-TUG1, PDGF-BB + si-TUG1 + anti-miR-NC, or PDGF-BB + si-TUG1 + anti-miR-216a-3p (ANOVA). The blots have been cropped. **C** Schematic illustration of the molecular mechanism of TUG1/miR-216a-3p/SMURF2 axis in PDGF-BB-stimulated HASMCs. *P < 0.05

To detect whether TUG1 sponged miR-216a-3p to regulate SMURF2 expression, HASMCs were introduced with si-NC, si-TUG1, si-TUG1 + anti-miR-NC, or si-AGAP2-AS1 + anti-miR-216a-3p, followed by stimulation with PDGF-BB. Transfection with si-TUG1 reduced SMURF2 mRNA and protein abundance in HASMCs stimulated with PDGF-BB, which was restored by co-introduction with miR-216a-3p inhibitor (Fig. 7A and B). Collectively, these data strongly supported the hypothesis that TUG1 could regulate HASMC proliferation and migration through modulating miR-216a-3p and SMURF2 expression (Fig. 7C).

## Discussion

Childhood asthma is one of the most common chronic diseases in childhood [12]. Accumulating evidence suggests that abnormal migration and proliferation of HASMCs play critical roles in the development of asthma [36]. PDGF-BB-caused the proliferation and migration of HASMCs is considered the main cause of airway wall thickening in asthma [24]. HASMCs stimulated with PDGF-BB were usually served as asthma model in vitro [5]*.* Therefore, blocking PDGF-BB-triggered the proliferation and migration of HASMCs represents a promising therapeutic strategy for treatment of childhood asthma.

Growing evidence has indicated that lncRNAs play pivotal roles in modulating HASMC proliferation and migration [2]. It has been reported that TUG1 is dysregulated in many diseases and implicated in diverse cellular processes, including differentiation, proliferation, migration, and apoptosis [33]. Moreover, TUG1 acts as a tumor-promoting lncRNA in multiple tumors, such as colorectal cancer [33], breast cancer [17], ovarian cancer [14], and cervical cancer [11]. In addition, TUG1 plays critical roles in regulating progression of multiple diseases, such as diabetic nephropathy [6], atherosclerosis [32], and myocardial infarction [32]. Besides, Lin et al. revealed that TUG1 expression was enhanced in the asthma rat model, and TUG1 facilitated ASMC proliferation and migration through regulation of miR-590-5p/FGF1 axis in asthma [18]. Agreement with this report, we observed that TUG1 expression was enhanced in whole blood samples of childhood asthma patients and HASMCs stimulated with PDGF-BB. Moreover, we uncovered that TUG1 knockdown limited the migration and proliferation of HASMCs under stimulation with PDGF-BB. These data disclosed that inhibition of TUG1 might be a promising strategy for treatment of childhood asthma.

LncRNAs have been elaborated to serve as ceRNAs or molecular sponges through binding to miRNAs to modulate the expression and function of downstream target mRNAs [30]. Bioinformatics analysis was performed to investigate whether TUG1 functioned as a miRNA sponge. We demonstrated that miR-216a-3p could bind with TUG1. It has been reported that some miRNAs, inducing miR-23b, miR-638 and miR-138, are aberrantly expressed and modulated HASMC proliferation and migration via targeting the 3’UTR of mRNAs [4, 19, 26]. As for miR-216a, it was identified to be lowly expressed in HASMCs from asthmatic patients and its overexpression limited cell growth and facilitated cell apoptosis [29], suggesting that miR-216a acted as a suppressor in asthma. Consistently, in our research, miR-216a-3p level was also declined in whole blood samples of childhood asthma patients and PDGF-BB-stimulated HASMCs. Moreover, our study revealed that miR-216a-3p inhibition abated the inhibiting impact of si-TUG1 on cell proliferation and migration in HASMCs stimulated with PDGF-BB. These results suggested that TUG1 exerted its functions in childhood asthma by sponging miR-216a-3p.

To clarify how miR-216a-3p affected the progression of childhood asthma, possible targets were predicted by starBase v2.0. We chose SMURF2 as a candidate target of miR-216a-3p for further investigation because it was associated with asthma progression. SMURF2 plays a vital role in modulation of allergic airway inflammation [31]. Wang et al. demonstrated that SMURF2 could interact with miR-485, and SMURF2 interference inhibited cell proliferation and increased apoptosis in ASMCs [27]. Herein, we uncovered that SMURF2 level was increased in whole blood samples of childhood asthma patients and PDGF-BB-stimulated HASMCs. Moreover, rescue experiments revealed that enforced expression of SMURF2 could abolish the suppressive impact of miR-216a-3p on proliferation and migration in HASMCs stimulated with PDGF-BB, suggesting that miR-216a-3p exerted its roles in childhood asthma through targeting SMURF2. Additionally, TUG1 positively regulated SMURF2 expression via sponging miR-216a-3p. Collectively, we found that TUG1 knockdown weakened HASMC proliferation and migration caused by PDGF-BB via regulation of miR-216a-3p/SMURF2 axis.

## Conclusions

Our study proved that TUG1 knockdown inhibited PDGF-BB-triggered HASMC cell proliferation and migration via regulating miR-216a-3p and SMURF2. The TUG1/miR-216a-3p/SMURF2 regulatory axis played a crucial role in HASMC proliferation and migration. These results might provide a new direction for childhood asthma treatment.

## Supplementary Information

**Additional file 1. **The concentrations of IL-4, IL-5 and IL-13 were increased in asthma patients. ELISA kits were used to detect the concentrations of IL-4, IL-5 and IL-13 in serum of childhood asthma patients and healthy subjects (ANOVA). *P < 0.05.

## Data Availability

The datasets used and/or analyzed during the current study are available from the corresponding author on reasonable request.

## Abbreviations

HASMCs: Human airway smooth muscle cells
lncRNAs: Long non-coding RNAs
TUG1: Taurine upregulated gene 1
ASMCs: Airway smooth muscle cells
kb: Kilobase
ceRNA: Competing endogenous RNA
miRNA: MicroRNA
SMURF2: Smad ubiquitin regulatory factor 2
UPP: Ubiquitin-proteasome pathway
PDGF-BB: Platelet-derived growth factor-two B chains
