# Supplementary figures and images for: Long non-coding RNA TUG1 promotes proliferation and migration in PDGF-BB-stimulated HASMCs by regulating miR-216a-3p/SMURF2 axis
Source: BMC Mol Cell Biol. 2021 Nov 8;22:56. doi: 10.1186/s12860-021-00396-0 (PMC8573901; doi:10.1186/s12860-021-00396-0)

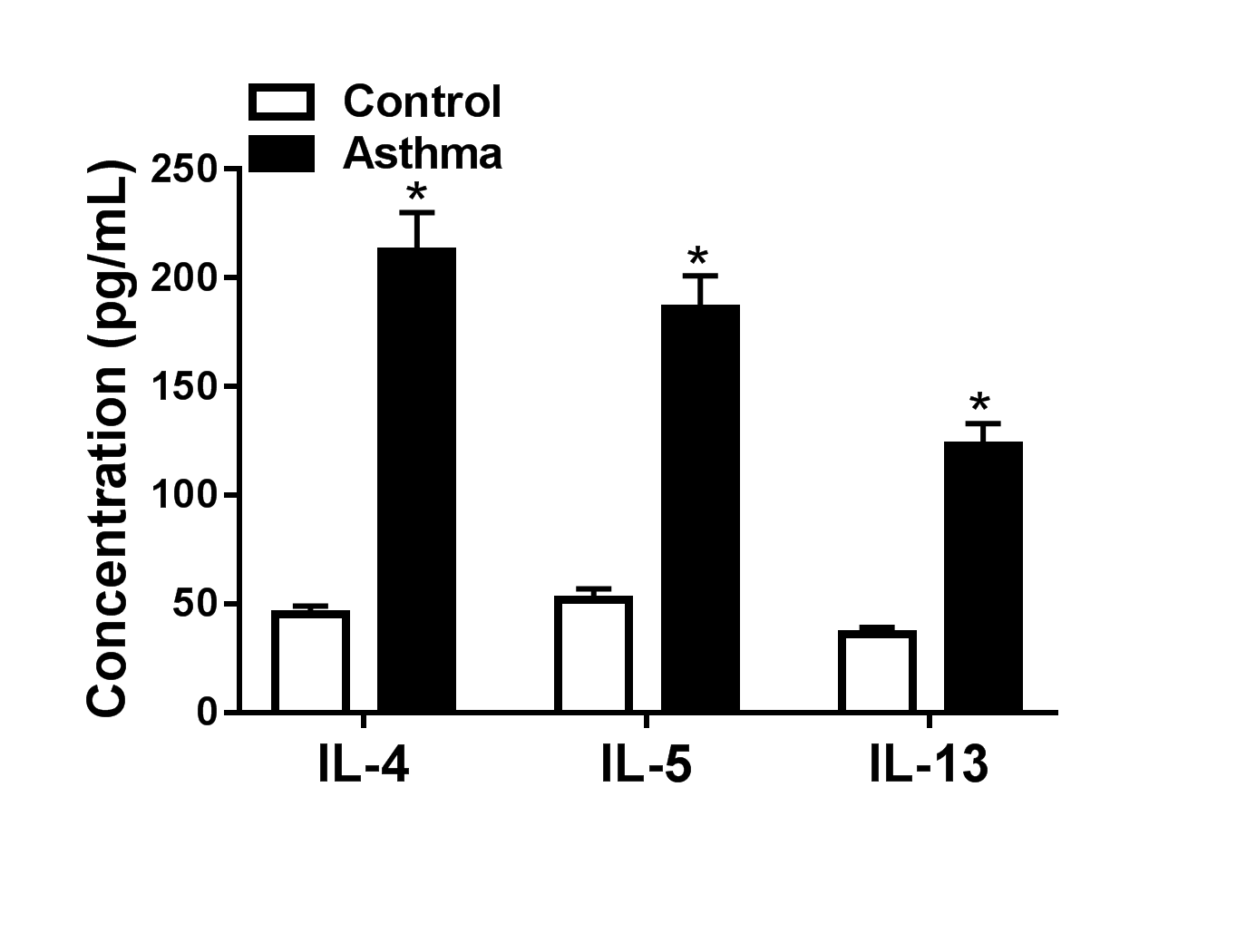

Supplement: Supplementary file 1 — Additional file 1. The concentrations of IL-4, IL-5 and IL-13 were increased in asthma patients. ELISA kits were used to detect the concentrations of IL-4, IL-5 and IL-13 in serum of childhood asthma patients and healthy subjects (ANOVA). *P < 0.05. [file 12860_2021_396_MOESM1_ESM.tif]
